# Cellulosic hydrocarbons production by engineering dual synthesis pathways in *Corynebacterium glutamicum*

**DOI:** 10.1186/s13068-022-02129-7

**Published:** 2022-03-15

**Authors:** Ying-Ying Xu, Ke-Jun Hua, Zhen Huang, Ping-Ping Zhou, Jing-Bai Wen, Ci Jin, Jie Bao

**Affiliations:** 1 State Key Laboratory of Bioreactor Engineering, East China University of Science and Technology, 130 Meilong Road, Shanghai, 200237 China; 2 College of Food and Biology Engineering, Henan University of Animal Husbandry and Economy, 6 Longzihu North Road, Zhengzhou, 450046 Henan China; 3 School of Chemical and Biological Engineering, Yichun University, 576 Xuefu Road, Yichun, 336000 Jiangxi China

**Keywords:** Hydrocarbons, Lignocellulose, *Corynebacterium glutamicum*, Dual synthesis pathways, Fatty acid decarboxylase OleT, Secretive expression

## Abstract

**Background:**

Lignocellulose provides the only practical carbohydrates feedstock for sustainable bioproduction of hydrocarbons as future alternative of fossil fuels. Production of hydrocarbons from lignocellulose is achieved by a biorefinery process chain including pretreatment to breakdown the crystalline structure for cellulase-catalyzed hydrolysis, detoxification of inhibitory compounds generated during pretreatment, enzymatic hydrolysis to fermentable monosaccharide sugars, and fermentation to hydrocarbon products. The major barriers on fermentative production of hydrocarbons from lignocellulose include two aspects: one is the inherent stress of pretreatment-derived inhibitors on microbial cells, the other is the toxicity of hydrocarbons to cell membranes. The microbial cell factory should be tolerant to both inhibitor stress and hydrocarbons toxicity.

**Results:**

*Corynebacterium glutamicum* was selected as the starting strain of hydrocarbons synthesis since it is well adapted to lignocellulose hydrolysate environment. The dual hydrocarbon synthesis pathways were constructed in an industrial *C. glutamicum* S9114 strain. The first pathway was the regular one in microalgae composed of fatty acyl-acyl carrier protein (fatty acyl-ACP) reductase (AAR) and aldehyde deformylating oxygenase (ADO) with fatty acyl-ACP as precursor. The second pathway was the direct decarboxylation of free fatty acid by fatty acid decarboxylase (OleT) using the rich fatty acids from the disruption of the transcriptional regulator *fasR* gene. The transmembrane transportation of hydrocarbon products was avoided by secretively expressing the fatty acid decarboxylase (OleT) to the extracellular space. The hydrocarbons generation from glucose reached 29.2 mg/L, in which the direct decarboxylation pathway contributed more than 70% of the total hydrocarbons generation, and the AAR–ADO pathway contributed the rest 30%.

**Conclusion:**

The dual hydrocarbon synthesis pathways (OleT and AAR–ADO pathways) were constructed in the inhibitors tolerant *C. glutamicum* S9114 strain for hydrocarbon production using lignocellulose feedstock as the starting feedstock. When corn stover was used for hydrocarbons production after dry acid pretreatment and biodetoxification, the hydrocarbons generation reached 16.0 mg/L. This study provided a new strategy for hydrocarbons synthesis using microbial cell factory suitable for lignocellulose feedstock.

**Supplementary Information:**

The online version contains supplementary material available at 10.1186/s13068-022-02129-7.

## Background

Current hydrocarbon fuels are dominantly produced from petroleum refining [1, 2]. The coming carbon-neutral economy requires the production of future aviation fuel and diesel in a sustainable way from renewable resources. Among all potential feedstocks, lignocellulose provides the only practical carbohydrates option for bioproduction of biofuels by its abundance and availability [3, 4].

Various microorganisms had been tested and engineered as microbial cell factories for hydrocarbons synthesis [5, 6]. The most acknowledged pathway is the decarbonylation of fatty acyl-ACP to fatty aldehydes by fatty acyl-ACP reductase (AAR) or the reduction of fatty acid by fatty acid reductase (FAR) [1, 7], then the fatty aldehydes are converted to hydrocarbons by aldehyde deformylating oxygenase (ADO) or aldehyde decarbonylase (ADS) [8, 9]. An alternative pathway is the direct decarboxylation of free fatty acids to terminal alkenes by fatty acid decarboxylase OleT [10], nonheme iron oxidase UndA [11], or fatty acid desaturase UndB [12] when abundant free fatty acids are available.

Pretreatment generally leads to the partial degradation of hemicellulose (mainly xylan) to furfural and cellulose to 5-hydroxymethyl furfural (HMF), and lignin to various phenolic compounds, along with other weak organic acids [13]. Biodetoxification fungus completely removes the most toxic inhibitors of furfural and HMF, as well as most of acetic acid and phenolics such as *p*-hydroxybenzaldehyde, vanillin, syringaldehyde, etc. However, the residual phenolics and acetic acid are not removed as completely as furfural and HMF because the extensive biodetoxification leads to the loss of xylose sugars. These residual inhibitors still show observable inhibitions to the cell growth and hydrocarbons synthesis metabolism of microorganisms such as *Escherichia coli* and most of the hydrocarbon synthesis strains. Furthermore, lipophilic hydrocarbons are toxic to cells due to the interference on cell membranes. The accumulation of hydrocarbons inside the cell increases the permeability and fluidity of the cell membrane and interferes with the function of membrane proteins, which in turn affects energy transfer and the stability of the cell membrane [14]. Previous studies showed that the microorganism *C. glutamicum* was unusually adaptive to lignocellulose hydrolysate environment by its strong tolerance to residual inhibitors of biodetoxifications and well utilization of rich vitamin B components in lignocellulose [15–20]. In this study, *C. glutamicum* was selected as the potential microbial cell factory [16, 18].

One of the uniqueness of *C. glutamicum* is that considerable free fatty acids are generated by *C. glutamicum* including oleic acid (C18:1), followed by palmitic acid (C16:0), and minor palmitoleic acid (C16:1) and stearic acid (C18:0) by the multi-enzyme complex fatty acid synthases (FASs) (one FAS-I modular and three FAS-II modules NCgl0281, NCgl0283 and NCgl0527) [21, 22]. The free fatty acids are accumulated and well maintained in extracellular space of *C. glutamicum* due to lack of β-oxidation pathway for fatty acid degradation, and lack of phosphatidic acid phosphatase (PAP) and diacylglycerol acyltransferase (DGAT) to triacylglyceride (microbial lipid) [23]. These advantages further suggest that *C. glutamicum* might be a favorable candidate of biorefinery hydrocarbons fermentation strains, though the cell membrane toxicity by hydrocarbons still exists.

We engineered an industrial *C. glutamicum* strain by constructing the dual hydrocarbon synthesis pathways in this study. One pathway was the regular algal pathway by fatty acyl-ACP reductase (AAR) and aldehyde deformylating oxygenase (ADO) with fatty acyl-CoA as precursor; the other pathway was the direct decarboxylation of fatty acid by fatty acids decarboxylase (OleT). The toxicity of the intracellularly generated hydrocarbons by transmembrane transportation was lessened by the secretive expression of OleT guided by a signal peptide. To increase the fatty acids substrate supply, the transcriptional regulator *fasR* gene was disrupted to increase C_16_ and C_18_ fatty acids as the additional substrate supply [24]. The resulting engineered *C. glutamicum* was applied for hydrocarbons fermentation using corn stover as feedstock for hydrocarbons production. This study provided a new strategy of metabolic engineering for hydrocarbons production using lignocellulose feedstock.

## Results

### Construction of AAR–ADO hydrocarbon synthesis pathway in *C. glutamicum*

**Fig. 1:**
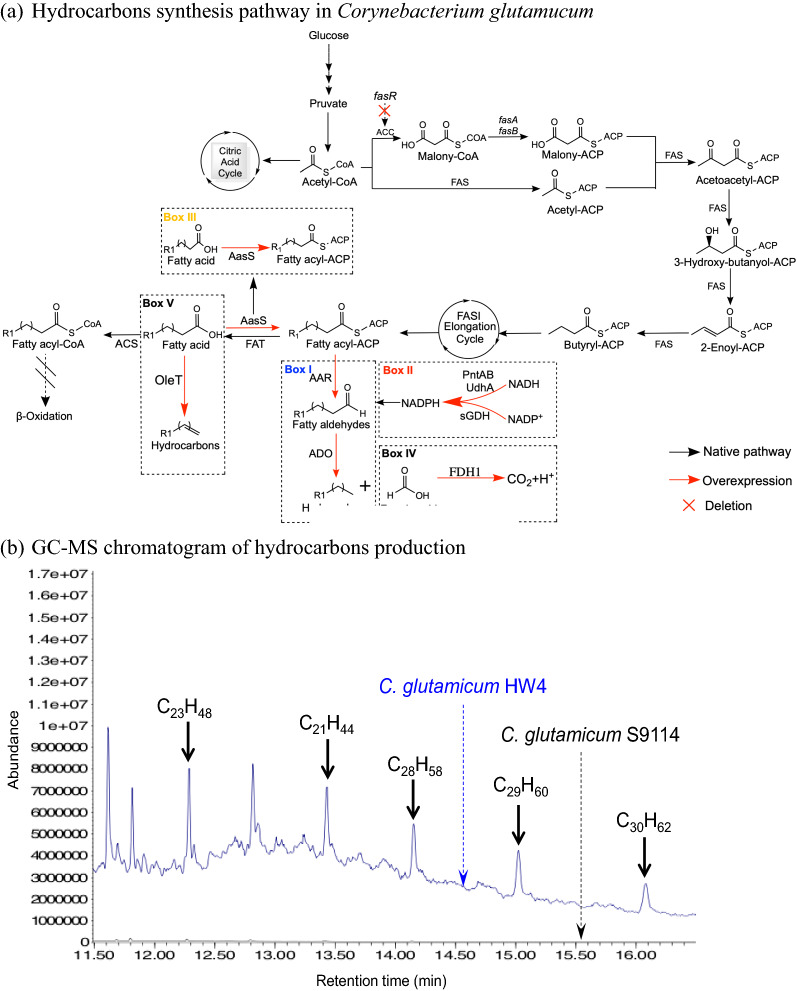

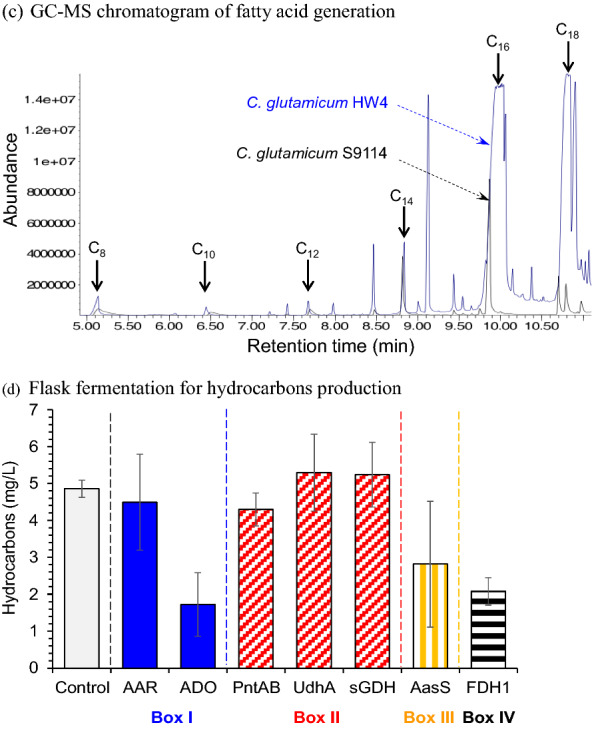
Hydrocarbons synthesis pathway in *Corynebacterium glutamicum* and construction of AAR–ADO pathway. **a** Overall hydrocarbons synthesis pathway in *C. glutamicum* S9114; black lines represent native pathways. Red lines represent overexpressing heterologous genes. Red cross represents the deletion of the gene. *ACP* acyl carrier protein, *AAR* acyl-ACP reductase, *ADH* aldehyde reductase, *ADO* aldehyde deformylating oxygenase, *ACC* acetyl CoA carboxylase, *FAS* fatty acid synthase, *FAT* fatty acyl-ACP thioesterase, *ACS* fatty acyl-CoA synthetase, *sGDH* glucose dehydrogenase, *FDH1* formic acid dehydrogenase, *AasS* fatty acyl-ACP synthetase, *OleT* fatty acid decarboxylase. **b** GC–MS chromatogram of hydrocarbons production in *C. glutamicum* S9114 (control) and HW4 (with AAR–ADO pathway). Shake-flask fermentation, 30 °C, 200 rpm, pH 7.0 maintained by adding 5 M NaOH. Abundance represented the response intensity of chromatogram peaks. *C. glutamicum* S9114 generated no hydrocarbons thus the peaks (black line) were not visible on the chromatogram; **c** metabolic modifications of *C. glutamicum* for hydrocarbons production chart. Fermentation parameters were similar to **b**

To construct the first AAR–ADO pathway from the fatty acyl-ACP substrate for hydrocarbons synthesis, the fragments of the lipoyl-ACP reductase gene *aar* (*synpcc7942_1594*) and the fatty aldehyde decarbonylase gene *ado* (*synpcc7942_1593*) from *Synechococcus elongatus* PCC7942 were synthesized and the expression plasmids were constructed; then the plasmid were introduced to *C. glutamicum* S9114 (Fig. 1a, Box I). The two genes *aar* (*synpcc7942_1594*) and *ado* (*synpcc7942_1593*) were expressed under the control of promoter H36 with different alignments (*C. glutamicum* ZW1, ZW2, and ZW3 in Table 1). The gene combination of *ado* and *aar* with the consensus SD sequence *aar*-*rbs*-*ado* (ZW2) showed the optimal hydrocarbon generation among the gene alignments. Then this gene fragment *aar*-*rbs*-*ado* was integrated into the location of the TetR-type transcriptional regulator *fasR* to generate a recombinant *C. glutamicum* HW4. Figure 1b shows that the typical alkene components C_21_H_44_, C_23_H_48_, C_28_H_58_, C_29_H_60_ and C_30_H_62_, among other hydrocarbon components, were detected in the fermentation broth of *C. glutamicum* HW4, indicating that the stable AAR–ADO hydrocarbon synthesis pathway had been established in genome scale.

**Table 1:** Strains and plasmids used

	Characteristics	Sources
Strains		
*E. coli* BL21	Host for plasmid construction	Lab stock
*Paecilomyces variotii* FN89	Biodetoxification fungus isolated by our lab	Lab stock
*C. glutamicum* S9114	Industrial strain	SIIM
*C. glutamicum* ZW1	*C. glutamicum* S9114 carrying pH36*-ado-aar*	This study
*C. glutamicum* ZW2	*C. glutamicum* S9114 carrying pH36*-ado-rbs*-*aar*	This study
*C. glutamicum* ZW3	*C. glutamicum* S9114 carrying pH36*-ado-*TacM-*aar*	This study
*C. glutamicum* HW4	*C. glutamicum* S9114 deleting *fasR* and carrying *aar* and *ado*	This study
*C. glutamicum* HW4-pH36*-aar*	*C. glutamicum* HW4 carrying pH36*-aar*	This study
*C. glutamicum* HW4-pH36*-ado*	*C. glutamicum* HW4 carrying pH36*-ado*	This study
*C. glutamicum* HW4-pH36-*pntAB*	*C. glutamicum* HW4 carrying pH36*-pntAB*	This study
*C. glutamicum* HW4-pH36-*udhA*	*C. glutamicum* HW4 carrying pH36*-udhA*	This study
*C. glutamicum* HW4-pH36-*sgdh*	*C. glutamicum* HW4 carrying pH36-*sgdh*	This study
*C. glutamicum* HW4-pH36-*aasS*	*C. glutamicum* HW4 carrying pH36-*aasS*	This study
*C. glutamicum* HW4*-*pH36-*fdh1*	*C. glutamicum* HW4 carrying pH36-*fdh*1	This study
*C. glutamicum* S9114-pH36-*oleT*_*JE*_	*C. glutamicum* S9114 carrying pH36-*oleT*_JE_	This study
*C. glutamicum* S9114-pH36-*oleT*_*MC*_	*C. glutamicum* S9114 carrying pH36-*oleT*_MC_	This study
*C. glutamicum* S9114-pH36-*NsoleT*_*JE*_	*C. glutamicum* S9114 carrying pH36-*NsoleT*_JE_	This study
*C. glutamicum* S9114-pH36-*RsoleT*_*JE*_	*C. glutamicum* S9114 carrying pH36-*RsoleT*_JE_	This study
*C. glutamicum* S9114-pH36-*NsoleT*_*MC*_	*C. glutamicum* S9114 carrying pH36-*NsoleT*_MC_	This study
*C. glutamicum* S9114-pH36-*RsoleT*_MC_	*C. glutamicum* S9114 carrying pH36-*RsoleT*_MC_	This study
*C. glutamicum* S9114-pH36-*NCgl1221*-oleT_MC_	*C. glutamicum* S9114 carrying pH36-*NCgl1221-oleT*_MC_	This study
*C. glutamicum* S9114-pH36-*NCgl1337*-*oleT*_MC_	*C. glutamicum* S9114 carrying pH36-*NCgl1337-oleT*_MC_	This study
*C. glutamicum* S9114-pH36-*PorB-oleT*_MC_	*C. glutamicum* S9114 carrying pH36-*porB-oleT*_MC_	This study
*C. glutamicum* S9114-pH36-*P*orC-oleT_MC_	*C. glutamicum* S9114 carrying pH36-*porC-oleT*_MC_	This study
*C. glutamicum* S9114-pEftu-NCgl1221-oleT_MC_	*C. glutamicum* S9114 carrying pEftu-*NCgl1221-oleT*_MC_	This study
*C. glutamicum* S9114-pEftu-NCgl1337-oleT_MC_	*C. glutamicum* S9114 carrying pEftu-*NCgl1337-oleT*_MC_	This study
*C. glutamicum* S9114-pEftu-*P*orB-oleT_MC_	*C. glutamicum* S9114 carrying pEftu-*porB-oleT*_MC_	This study
*C. glutamicum* S9114-pEftu-*P*orC-oleT_MC_	*C. glutamicum* S9114 carrying pEftu-*porC-oleT*_MC_	This study
*C. glutamicum* HW4-pH36-oleT_JE_	*C. glutamicum* HW4 carrying pH36-*oleT*_JE_	This study
*C. glutamicum* HW4-pH36-oleT_MC_	*C. glutamicum* HW4 carrying pH36-*oleT*_MC_	This study
*C. glutamicum* HW4-pH36-NsoleT_JE_	*C. glutamicum* HW4 carrying pH36-*NsoleT*_JE_	This study
*C. glutamicum* HW4-pH36-RsoleT_JE_	*C. glutamicum* HW4 carrying pH36-R*soleT*_JE_	This study
*C. glutamicum* HW4-pH36-NsoleT_MC_ (*C. glutamicum* HW5)	*C. glutamicum* HW4 carrying pH36-*NsoleT*_MC_	This study
*C. glutamicum* HW4-pH36-RsoleT_MC_ (*C. glutamicum* HW6)	*C. glutamicum* HW4 carrying pH36-*RsoleT*_MC_	This study
Plasmids		
pK18mobsacB	Mobilizable vector in *C. glutamicum*, kanamycin resistance, sacB	Wang et al. [17]
pH36mob	Overexpression vector, kanamycin resistance	Lab stock
pEftumob	Insert promoter P*eftu* at the back of promoter P*trc* in pTRCmob	Lab stock
pH36-*ado-aar*	pH36mob carrying *aar* and *ado*	This study
pH36-*ado-rbs-aar*	pH36mob carrying *aar*, *rbs* and *ado*	This study
pH36-*ado-*TacM-*aar*	pH36mob carrying ado under H36 control and *aar* under TacM control	This study
pH36-*aar*	pH36mob carrying *aar*	This study
pH36-*ado*	pH36mob carrying *ado*	This study
pH36-*pntAB*	pH36mob carrying *pntAB*	This study
pH36-*sgdh*	pH36mob carrying *sgdh*	This study
pH36-*udhA*	pH36mob carrying *udhA*	This study
pH36-*aasS*	pH36mob carrying *aasS*	This study
pH36-*fdh1*	pH36mob carrying *fdh1*	This study
pH36-*oleT*_JE_	pH36mob carrying *oleT*_JE_	This study
pH36-*oleT*_MC_	pH36mob carrying *oleT*_MC_	This study
pH36-*NsoleT*_JE_	pH36mob carrying *oleT*_JE_ with signal peptide of *Ncgl1289*	This study
pH36-*RsoleT*_JE_	pH36mob carrying *oleT*_JE_ with signal peptide of *RS04950*	This study
pH36-*NsoleT*_MC_	pH36mob carrying *oleT*_MC_ with signal peptide of *Ncgl1289*	This study
pH36-*RsoleT*_MC_	pH36mob carrying *oleT*_MC_ with signal peptide of *RS04950*	This study
pH36-*NCgl1221-oleT*_MC_	pH36mob carrying *oleT*_MC_ with signal peptide of *Ncgl1221*	This study
pH36-*NCgl1337-oleT*_MC_	pH36mob carrying *oleT*_MC_ with signal peptide of *Ncgl1337*	This study
pH36-*PorB-oleT*_MC_	pH36mob carrying *oleT*_MC_ with signal peptide of *PorB*	This study
pH36-*PorC-oleT*_MC_	pH36mob carrying *oleT*_MC_ with signal peptide of *PorC*	This study
pEftu-*NCgl1221-oleT*_MC_	pH36-*NCgl1221-oleT*_MC_ with H36 promoter replaced by Eftu promoter	This study
pEftu-*NCgl1337-oleT*_MC_	pH36-*NCgl1337-oleT*_MC_ with H36 promoter replaced by Eftu promoter	This study
pEftu-*PorB-oleT*_MC_	pH36-*PorB-oleT*_MC_ with H36 promoter replaced by Eftu promoter	This study
pEftu-*PorC-oleT*_MC_	pH36-*PorC-oleT*_MC_ with H36 promoter replaced by Eftu promoter	This study
pK18-ΔfasR-*aar-rbs-ado*	Plasmid for fasR knockout in the genome and carrying *aar*, *rbs* and *ado*	This study

The *fasR* disruption by the *aar*-*rbs*-*ado* cluster also led to the excessive generation of C_16_ and C_18_ fatty acids. The fatty acids generation by the parental *C. glutamicum* S9114 was about ~ 10 mg/L fermentation broth by GC–MS detection with dodecane as the standard. The disruption of *fasR* in *C. glutamicum* HW4 resulted in the fatty acid generation up to 103 mg/L, approximately one order of magnitude greater than that by the parental strain (Fig. 1c).

To increase the hydrocarbons production of *C. glutamicum* HW4, a systematic metabolic engineering was performed (Fig. 1a) including (i) separately overexpressing *aar* and *ado* in plasmids pH36mob to increase the copy numbers (Fig. 1a, Box I); (ii) expression of NADPH reductase genes *pntAB*, *udhA* and *sgdh* to increase the NADPH supply (Fig. 1a, Box II); (iii) expression of fatty acyl-ACP synthase AasS from *Vibrio harveyi* B392 to convert free fatty acids to fatty acyl-ACP (Fig. 1a, Box III) [25], and (iv) expression of formic acid dehydrogenase FDH1 from *S. cerevisiae* S288C to degrade the byproduct formic acid (Fig. 1a, Box IV). However, these efforts showed no improvements or even negative results (Fig. 1d), except that the enhancement of NADPH supply by overexpression of *udhA* and *sgdh* genes (encoding UdhA and sGDH, respectively) with ~ 8% increase of hydrocarbons production.

### Constructing the fatty acid decarboxylation pathway in *C. glutamicum* and the secretive expression

**Fig. 2:**
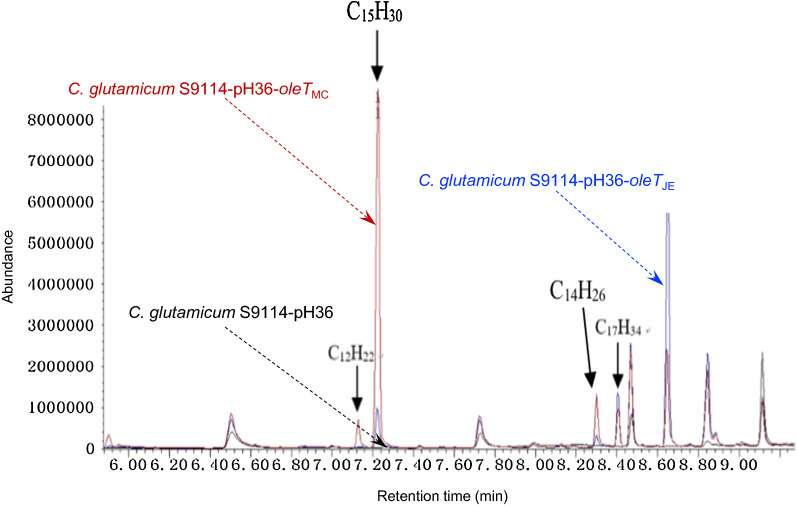
GC–MS result of hydrocarbon production via different fatty acid decarboxylases in *C. glutamicum* S9114. Shake-flask fermentation at 30 °C, 200 rpm, pH 7.0 maintained by adding 5 M NaOH

To increase the hydrocarbons production by *C. glutamicum*, the second pathway was constructed by direct decarboxylation of free fatty acid. Free fatty acids accumulation is the unique phenomenon of *C. glutamicum* because of the lack of lack of β-oxidation pathway for fatty acid degradation, as well as the lack of phosphatidic acid phosphatase (PAP) and diacylglycerol acyltransferase (DGAT) to produce triacylglyceride (microbial lipid) [21]. The direct decarboxylation pathway of free fatty acids was constructed by overexpression of the *oleT* gene encoding the fatty acid decarboxylase OleT (Fig. 1a, Box V). Two fatty acid decarboxylase genes, *oleT*_JE_ from *Jeotgalicoccus* sp. ATCC 8456 and *oleT*_MC_ from *Macrococcus caseolyticus* WP_041635889.1 [10, 26], were selected, synthesized, and heterologously expressed in *C. glutamicum* S9114. The di-alkenes of C_12_H_22_ and C_14_H_26_, as well as the mono-alkenes of C_15_H_30_ and C_17_H_34_, were produced by the overexpression of the two *oleT* genes (Fig. 2). The greater hydrocarbons generation of *oleT*_MC_ expression indicates OleT_MC_ was more adaptive for fatty acid decarboxylation in *C. glutamicum* S9114.

**Fig. 3:**
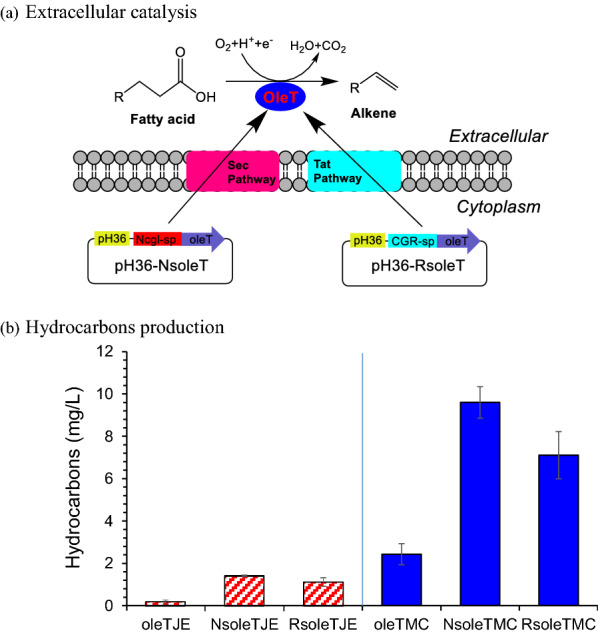
Secretive expression of fatty acid decarboxylase and hydrocarbon synthesis in *C. glutamicum* S9114. **a** Illustration of concept; **b** hydrocarbons production. oleT_JE_, oleT_MC_ represent *C. glutamicum* S9114 with the overexpression of corresponding genes; NsoleT_JE_, NsoleT_MC_ represent *C. glutamicum* S9114 with the secretive expression of *oleT*_JE/MC_ through Sec pathway; RsoleT_JE_, RsoleT_MC_ represent *C. glutamicum* S9114 with the secretive expression of *oleT*_JE/MC_ through Tat pathway. Shake-flask fermentation, 30 °C, 200 rpm, pH 7.0 maintained by adding 5 M NaOH

To avoid the cell membrane damage by the transmembrane transportation of hydrocarbons, the extracellular decarboxylation of fatty acids into hydrocarbons were designed by the secretive expression of OleT (Fig. 3a). Two secretory pathways were tested, one was the Sec pathway to secret the unfolded proteins by the peptide *Ncgl1289* from *C. glutamicum* ATCC13032 [27], the other was the Tat pathway to secret the folded proteins by *cgR_0494* from *C. glutamicum* S9114 [28–30]. Each of the signal peptide genes was ligated with *oleT*_JE_ and *oleT*_MC_ by overlap PCR, then inserted into the expression plasmids and introduced to *C. glutamicum* S9114 to obtain four recombinants, S9114-pH36-*NsoleT*_JE_, S9114-pH36-*RsoleT*_JE_, S9114-pH36-*NsoleT*_MC_ and S9114-pH36-*RsoleT*_MC_. Both the SDS-PAGE and the Western blotting did not show the clear protein bands due to the high fatty acids content for protein extraction in the fermentation broth. Figure 3b shows that the secretive expression of OleT significantly improved the hydrocarbons generation. Among the hydrocarbons produced, the secretive expression of OleT_MC_ by the Sec pathway (S9114-pH36-NsoleT_MC_) showed approximately fourfolds greater hydrocarbons (9.6 mg/L) than the intracellular expression (S9114-pH36-OleT_MC_, 2.4 mg/L), and approximately 36% more hydrocarbons than the secretive expression of OleT_MC_ by the Tat pathway (S9114-pH36-RsoleT_MC_, 7.1 mg/L). The results suggest that the cell damage was partially relieved by the secretion of decarboxylase and considerable hydrocarbons were generated in the extracellular space.

**Fig. 4:**
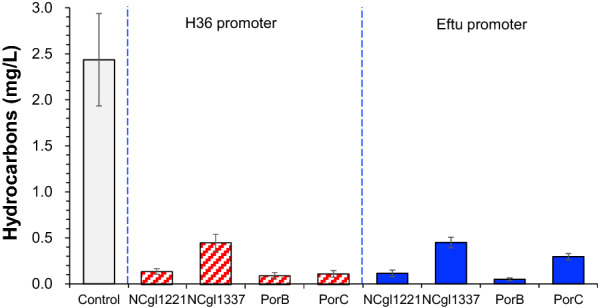
Hydrocarbons production of displaying fatty acid decarboxylase OleT_MC_ on the cell surface under the control of H36 promote and Eftu promoter. Control represents *C. glutamicum* S9114 with the overexpression of *oleT*_MC_. Fermentations were carried out in shake flask, 30 °C, 200 rpm, pH 7.0 maintained by adding 5 M NaOH

Cell surface display of the OleT expression was also tried by expressing the anchor proteins NCgl1221, NCgl1337, PorB and PorC from *C. glutamicum* S9114. Unfortunately, the results show no observable improvement or even suppression by the surface display expression of OleT under the control of the promotor Eftu and H36 (Fig. 4).

### Dual pathways construction for hydrocarbons synthesis in *C. glutamicum*

**Fig. 5:**
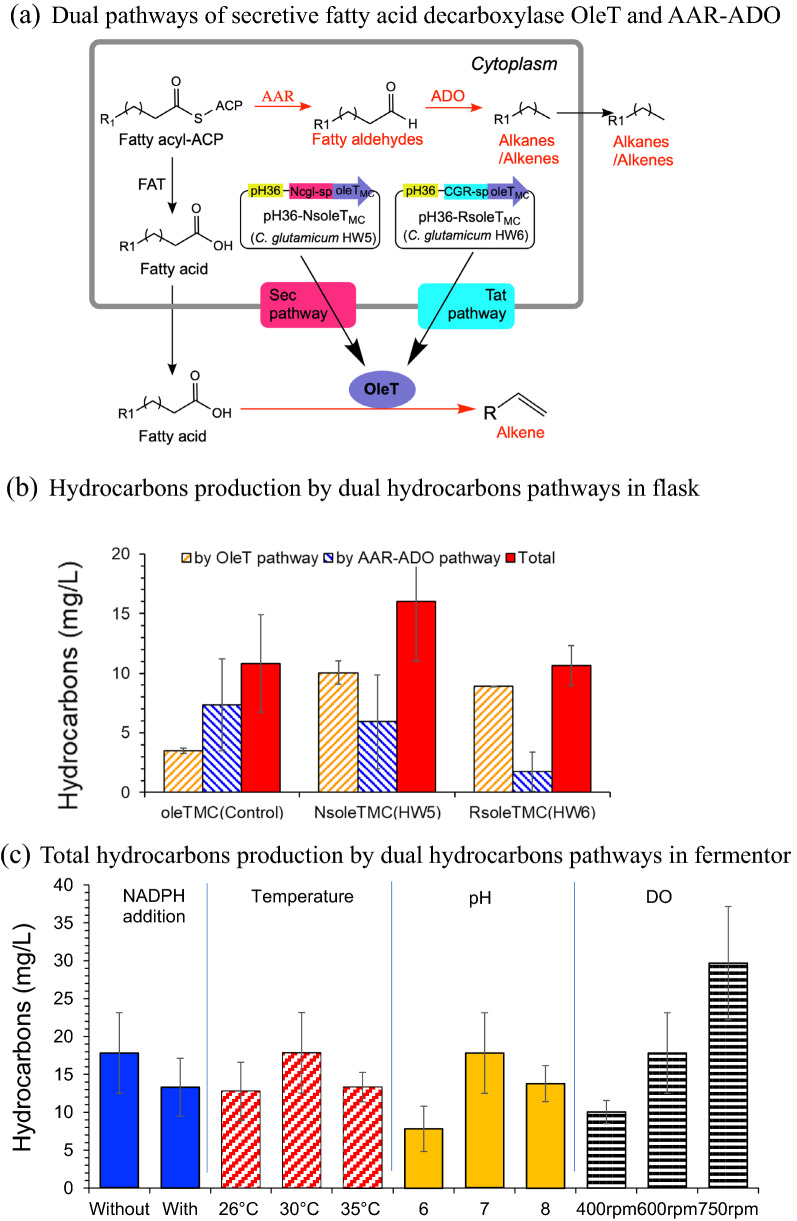
Hydrocarbons production by dual synthesis pathways in *C. glutamicum*. **a** Secretive expression of fatty acid decarboxylase OleT_MC_ in *C. glutamicum* HW4. **b** Hydrocarbons production by dual synthesis pathways in *C. glutamicum*. HW5 indicates *C. glutamicum* HW4 with the secretive expression of *oleT*_MC_ through Sec pathway and HW6 indicates *C. glutamicum* HW4 with the secretive expression of *oleT*_MC_ through Tat pathway. Fermentation was carried out in shake flask, 30 °C, 200 rpm. pH was maintained at 7.0 by adding 5 M NaOH. **c** Improved hydrocarbons production by optimizing the culture conditions. Fermentation was carried out in a 1-L fermentor. The medium was mentioned in “Materials and methods”. The basic fermentation conditions were 30 °C, pH 7 and 600 rpm. When one of the fermentation parameters was changed for optimization, the others remained the same

The two secretive overexpression of OleT_MC_ by the Sec secretive pathway and the Tat secretive pathway were expressed in *C. glutamicum* HW4 (carrying the AAR–ADO pathway in genome scale) to give the two recombinants *C. glutamicum* recombinants, HW5 and HW6, respectively (Fig. 5a).

Figure 5b shows that both the recombinants *C. glutamicum* HW5 and HW6 significantly enhanced the generation of both the di-alkenes (C_12_H_22_ and C_14_H_26_) and the mono-alkenes (C_15_H_30_ and C_17_H_34_). The hydrocarbons generation by OleT pathway in *C. glutamicum* HW5 (Sec secretive pathway) (10.1 mg/L) were 1.9 times greater than the control (without the secretive expression HW4-pH36-OleT_MC_, 3.5 mg/L), and 13% higher than that of *C. glutamicum* HW6 (Tat secretive pathway, 8.9 mg/L). The total hydrocarbons production by the dual pathways of AAR–ADO and the OleT_MC_ in *C. glutamicum* HW5 reached 16.0 mg/L.

The optimal fermentation parameters of *C. glutamicum* HW5 with the dual hydrocarbons synthesis pathways were examined in bioreactors with automatic pH control and dissolved oxygen input using glucose as carbon resource (Fig. 5c). The results show that the hydrocarbons production was not affected by NADPH addition, and 30 °C and pH 7.0 were suitable for cell growth and hydrocarbons production; the hydrocarbons generation increased with increasing oxygen transfer rate by varying the stirring rate. At the proper fermentation conditions (30 °C, pH 7.0 and 750 rpm), totally 29.2 mg/L of hydrocarbons were produced, including 14.5 mg/L of di-alkenes and 7.3 mg/L of mono-alkenes by *C. glutamicum* HW5.

**Fig. 6:**
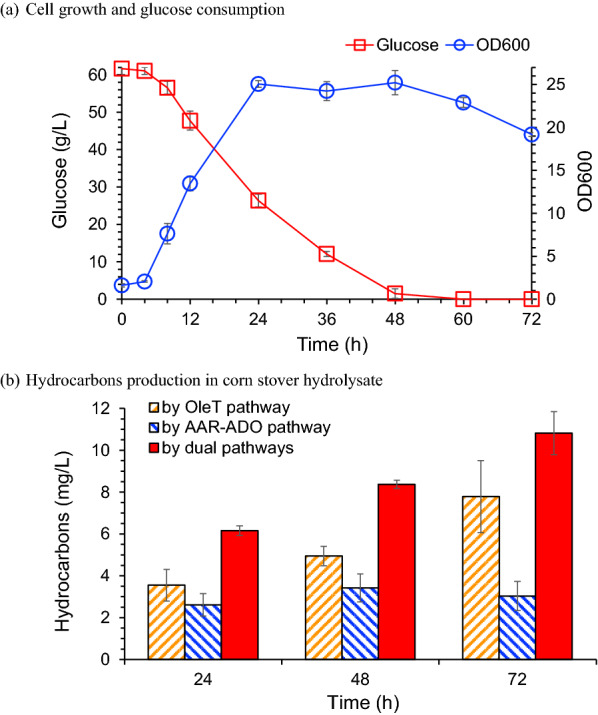
Hydrocarbons fermentation by *C. glutamicum* HW5 using corn stover hydrolysate. Fermentation was carried out in a 3-L fermentor, 30 °C, 600 rpm and 1.4 vvm of aeration. pH was maintained at 7.0 by adding 5 M NaOH and 2 M H_2_SO_4_. Corn stover hydrolysate was prepared by hydrolyzing the biodetoxified corn stover

Corn stover was used as carbohydrates feedstock for hydrocarbon production by *C. glutamicum* HW5 (Fig. 6a). Corn stover hydrolysate was prepared by enzymatically hydrolyzing 15% (w/w) of the dry acid pretreated and biodetoxified corn stover with the addition of 5 g/L (NH_4_)_2_SO_4_, 1 g/L KH_2_PO_4_, 1 g/L K_2_HPO_4_ and 0.25 g/L MgSO_4_. The total hydrocarbons production by the dual pathways in *C. glutamicum* HW5 reached 10.8 mg/L, in which the OleT_MC_ pathway generated 7.8 mg/L and the AAR–ADO pathway generated 3.0 mg/L of hydrocarbons.

The hydrocarbons generated from the two pathways shared different carbon chain lengths. The carbon number of hydrocarbons from the OleT pathway was less than 20 and the retention time on GC–MS chromatograph was between 7–8 min. The carbon number from the AAR–ADO pathway was greater than 20 and the retention time on GC–MS was greater than 10 min (Fig. 6b). The hydrocarbons generation could be translated to 0.205 mg hydrocarbons/g cellulose in corn stover.

Hydrocarbons production from biomass has been the focus of metabolic engineering and various microorganisms had been engineered as microbial cell factories for hydrocarbons synthesis. This study used *C. glutamicum* as the hydrocarbons producing strain by its two inherent properties: inhibitors tolerance and free fatty acid generation. The dual hydrocarbons synthesis pathways were constructed and 10.8 mg/L of hydrocarbons was generated when corn stover was used, in which the OleT pathway contributed more than 70% of the total generation of hydrocarbons. The high residual fatty acids level indicated a further upgrading potential of hydrocarbons production by further metabolic engineering.

One of the uncertainties of hydrocarbons production is the less accurate calculation of hydrocarbons content based on the peak areas of hydrocarbons generation of GC–MS chromatogram due to the high and irregular fatty acids peaks on the baseline of GC–MS chromatogram. Due to the high boiling point of fatty acid and non-volatility, the accuracy of hydrocarbons measurement was negatively affected. Highly possible, the hydrocarbons contents were under-estimated because of the merge of hydrocarbons peaks with fatty acids peaks. We tried to measure the fatty acid content using GC–MS and eliminate the fatty acid content from hydrocarbon components for an accurate measurement. However, the high boiling points of fatty acids led to the incomplete volatilization of fatty acids and carbon residues accumulation in GC column (thus damaged the column). We also tried the esterification of the fatty acids in the samples, but this procedure caused a heavy loss of hydrocarbons and the accuracy of hydrocarbon measurement was even worse. A more accurate analysis method is under investigation to determine hydrocarbons contents with the existence of high free fatty acids in the fermentation broth.

## Conclusion

The dual hydrocarbon synthesis pathways (OleT and AAR–ADO pathways) were constructed in the inhibitors tolerant *C. glutamicum* S9114 strain for hydrocarbon production using lignocellulose feedstock as the starting feedstock. The first one is the regular AAR–ADO pathway and the second is the fatty acid decarboxylation pathway from fatty acids by taking advantage of free fatty acid generation of *C. glutamicum*. The fatty acid decarboxylation pathway was further enhanced significantly by secretive expression of fatty acid decarboxylase OleT and performed the extracellular catalysis by secreted OleT enzyme. The hydrocarbons generation from glucose reached 29.2 mg/L, in which the direct decarboxylation pathway contributed more than 70% of the total hydrocarbons generation, and the AAR–ADO pathway contributed the rest 30%. When corn stover was used for hydrocarbons production after dry acid pretreatment and biodetoxification, the hydrocarbons generation reached 16.0 mg/L. This study provided a new strategy for hydrocarbons synthesis using microbial cell factory suitable for lignocellulose feedstock. The high residual fatty acids level indicated a further upgrading potential of hydrocarbons production by further metabolic engineering.

## Discussion

Although the major inhibitors (furfural, 5-hydroxymethylfurfural, acetic acid) generated during pretreatment were removed by biodetoxification, the residual phenolic compounds still showed considerable stress on fermentation strains. In this study, we selected a high robust *C. glutamicum* as microbial cell factory for synthesis of hydrocarbons and the hydrocarbon products were successfully synthesized by the engineered *C. glutamicum* using corn stover feedstock after dry biorefinery processing. To improve the low hydrocarbons synthesis efficiency of *C. glutamicum*, two hydrocarbon synthesis pathways were constructed, the first hydrocarbon synthesis pathway of AAR–ADO originates from cyanobacteria, and the second fatty acid decarboxylation pathway utilizes the unique and rich free fatty acid substrates.

Due to the knockout of the *fasR* gene, the engineered *C. glutamicum* accumulated considerably high fatty acids in the extracellular environment. We expressed the fatty acid decarboxylase and secreted the enzyme into the extracellular space (fermentation broth) to conduct the fatty acid decarboxylation reaction in extracellular way. This method avoided the accumulation of hydrocarbons inside the cells and the lessened the toxicity on membrane integrity, resulting in a threefold higher production of hydrocarbons than that of the control strain. We also were found there was still high level of free fatty acids in the fermentation broth after the decarboxylation, suggesting that the fatty acid decarboxylase activity was not enough to convert the fatty acids to the hydrocarbons completely. The further improvement on catalytic efficiency of fatty acid decarboxylase is required. The major contribution of this study is the dual pathways of hydrocarbons synthesis with great potentials for future engineering of microbial cell factory with strong lignocellulose derived inhibitors tolerance.

## Materials and methods

### Strains and media

Strains and plasmids used in this study are listed in Table 1.

*Escherichia coli* BL21 was used for plasmid construction and cultured in LB medium. *C. glutamicum* S9114 was used as the starting strain and the genome sequence referred in NCBI with the access number NZ_AFYA01000018. *C. glutamicum* was cultured in CM2B medium (yeast extract 10 g/L, peptone 10 g/L, NaCl 10 g/L). The CGXII-NL medium for hydrocarbons fermentation contained 60 g/L glucose, 1.0 g/L (NH_4_)_2_SO_4_, 2.5 g/L urea, 1.0 g/L KH_2_PO_4_, 1.0 g/L K_2_HPO_4_, 42 g/L M_O_PS, 0.25 g/L MgSO_4_, 0.01 g/L CaCl_2_, 0.01 g/L FeSO_4_·7H_2_O, 0.01 g/L MnSO_4_·H_2_O, 0.001 g/L ZnSO_4_·7H_2_O, 0.0002 g/L CuSO_4_·5H_2_O, 0.00002 g/L NiCl_2_·6H_2_O, 0.0002 g/L biotin, 0.0005 g/L thiamin, 0.03 g/L PCA. 50 μg/mL of kanamycin was added into the media if needed.

### Plasmids and recombinants construction

The primers used for plasmids construction are shown in Additional file 1: Table S1. The fragments of *aar*, *ado*, *sgdh*, *aasS*, *fdh1*, *oleT*_JE_ and *oleT*_MC_ genes were synthesized by Shanghai Generay Biotech, Shanghai, China. The *pntAB* and *udhA* genes were amplified from *E. coli* BL21 genome. These fragments were then constructed into the expression vectors pH36mob and pEftumob separately by digestion-ligation or in-fusion cloning. The fragments of *aar-ado*, *ado-rbs-aar* and *ado-*TacM*-aar* were obtained by overlapping the corresponding fragments and then inserted into the expression vector pH36mob, resulting in several plasmids pH36-*aar-ado*, pH36-*ado-rbs-aar*, and pH36-*ado-*TacM*-aar*.

The signal peptide sequences of *Ncgl1289* and *cgR_0949* were amplified from *C. glutamicum* ATCC 13032 and *C. glutamicum* S9114. Then the signal peptide sequences of *Ncgl1289* and the fragment of *oleT*_JE_ gene were overlapped together and inserted into pH36mob, resulting pH36-*NsoleT*_JE_. Plasmids pH36-*NsoleT*_MC_, pH36-*RsoleT*_JE_ and pH36-*RsoleT*_MC_ were obtained in the same way. Plasmids were constructed similarly by fusing membrane protein sequences of *Ncgl1337*, *Ncgl1221*, *porB*, and *porC* in front of *oleT*_MC_ for expression of *oleT*_MC_ on the cell surface under the control of the promotor Eftu and H36. All the above overexpression plasmids were verified via sequencing analysis and then transformed into *C. glutamicum* by electroporation. The recombinant strains grown on plates with kanamycin resistance were verified by colony PCR.

The up- and down-fragments of *fasR* gene were cloned from *C. glutamicum* S9114, and then inserted into pK18mobsacb. The fragment *aar-rbs-ado* was inserted between the up- and down-fragments of *fasR* gene, resulting in the pK18-ΔfasR-*aar-rbs-ado* plasmid. This plasmid was verified by sequencing analysis and transformed into *C. glutamicum* by electroporation. The correct recombinant mutant was isolated through two rounds of homologous recombination and verified by colony PCR [31].

### Lignocellulose feedstock and biorefinery processing

Corn stover was harvested from Nanyang, Henan, China, in fall 2020. The raw biomass was air dried and milled, and then pretreated using the dry acid pretreatment method [32, 33]. Acid pretreatment was operated according to the protocols in [34–38]. A 20-L helical ribbon impeller-driven reactor was fed with 1200 g of corn stover (dry base) and 500–600 g of sulfuric acid solution to the dry solid weight to the acid liquid weight of 2:1. The corn stover and acid solution were co-currently fed into the reactor and stirred for 3 min at 50 rpm. The hot steam was then jetted into the reactor and maintained at 175 °C for 5 min. The pretreated corn stover solids were discharged from the bottom outlet port of the reactor without free wastewater generation, then briefly milled to move the extra-long fibers. The acid catalyst usage was adjusted according to the method previously described [39].

The solid-state biodetoxification was conducted in a 15-L bioreactor. The spore suspension of *Amorphotheca resinae* ZN1 was inoculated to the freshly pretreated corn stover solids and cultured at 30 °C for 48 h. Then the seed was inoculated into pretreated corn stover solids at 10% (w/w) mass ratio, and incubated at 30 °C for 36–48 h with the aeration rate of 1 vvm (air volume per culture volume per min). The brief stirring was conducted at 50 rpm every 12 h [33, 40]. The corn stover hydrolysate (CSH) was prepared by hydrolyzing the biodetoxified corn stover [16]. The duration of all fermentations was 72 h. All fermentations were carried out in triplicate, and the error bars were indicated by the standard derivations of three biological replicates.

### Hydrocarbon fermentation

The seed culture was prepared as described in our previous study [16]. The shake-flask fermentation was conducted by inoculating the seed culture at 5% (v/v) inoculum ratio in 250-mL shake flasks containing 30 mL CGXII medium at 30 °C, 200 rpm. The pH was maintained at 7.0 by adding 5 M NaOH.

The bioreactor fermentation was conducted in a 1-L fermenter at 30 °C, 600 rpm and 1.4 vvm aeration, pH 7.0 by adding 2 M H_2_SO_4_ and 5 M NaOH automatically. The seed culture was inoculated into 600 mL of the fermentation medium at 10% (v/v) inoculum ratio. The cellulosic hydrocarbons fermentation was carried out in a 3-L fermenter containing 800 mL 15% (w/w) solids content corn stover hydrolysate. The other fermentation conditions were the same as mentioned above.

### Hydrocarbon extraction and quantification

Hydrocarbons were extracted from 60 mL fermentation broth using 30 mL the mixture of methanol and chloroform (the ratio of methanol/chloroform was 2:1) for 24 h and then centrifuged at 10,000 rpm for 10 min. The solvent layer on the bottom of the mixture was rotary-evaporated to remove the solvent and the hydrocarbons obtained were re-dissolved by adding 1 mL of chloroform to obtain the samples for hydrocarbons measurement.

Samples were analyzed by Agilent 6890 GC–MS (Agilent Technologies, Santa Clara, CA, USA) with HP-5-MS column. The initial temperature was 50 °C and maintained for 2 min, then ramped up to 80 °C at a rate of 15 °C/min and held at 80 °C for 3 min, after that the temperature was ramped up to 280 °C at a rate of 15 °C/min and held at 280 °C for 8 min. The flow rate of the carrier gas helium was 1 mL/min. NIST MS SEARCH 2.0 library was used for qualitative analysis, and the matching degree of samples and standard products reached more than 95%. The internal calibration was 100 mg/L dodecane (C_12_H_26_) and the concentration of the hydrocarbons was calculated according to the ratio of the chromatographic peak area.

## Supplementary Information

**Additional file 1: Table S1.** Primers used in this study.

## Data Availability

All data generated or analyzed during this study are included in this article. If additional information is needed, please contact the corresponding author.
