# Supplementary material for: Cellulosic hydrocarbons production by engineering dual synthesis pathways in Corynebacterium glutamicum
Source: Biotechnol Biofuels Bioprod. 2022 Mar 15;15:29. doi: 10.1186/s13068-022-02129-7 (PMC8922798; doi:10.1186/s13068-022-02129-7)
Supplement: Supplementary file 1 — Additional file 1: Table S1. Primers used in this study. [file 13068_2022_2129_MOESM1_ESM.docx]

**ADDITIONAL FILE 1**

**Table S1**. Primers used in this study.

| **Primers** | **Sequences (5’-3’)** | **Restriction enzymes** |
| --- | --- | --- |
| NsoleT_JE_-F1  NsoleT_JE_-R1  NsoleT_JE_-F2  NsoleT_JE_-R2  RsoleT_JE_-F1  RsoleT_JE_-R1  RsoleT_JE_-F2  RsoleT_JE_-R2  NsoleT_MC_-F1  NsoleT_MC_-R1  NsoleT_MC_-F2  NsoleT_MC_-R2  RsoleT_MC_-F1  RsoleT_MC_-R1  RsoleT_MC_-F2  RsoleT_MC_-R2  NCgl1221-F1  NCgl1221-R1  NCgl1221-F2  NCgl1221-R2  NCgl1337-F1  NCgl1337-R1  NCgl1337-F2  NCgl1337-R2  PorB-F1  PorB-R1  PorB-F2  PorB-R2  PorC-F1  PorC-R1  PorC-F2  PorC-R2  NCgl1221MC-F  NCgl1221MC-R  NCgl1337MC-F  NCgl1337MC-R  PorBMC-F  PorBMC-R  PorCMC-F  PorCMC-R | CCTCTAGAATGAAATATGAATTTAATAATAGATTCCGAAC  CTTAAGTGTTGCCATAAAGAGCTCCTGATCATGTAGGTGTC  GATCAGGAGCTCTTTATGGCAACACTTAAGAGGGGATAAG  GCAGGTCGACTTAGTGATGGTGATGGTGATGTGTTCTGTCTACAACTTCGCGAACA  CCTCTAGAATGCAAATAAACCGCCGAGG  TCCCTCTTAAGTGTTGCCATTGCTCCCTGGGCGTTG  GCCAACGCCCAGGGAGCAATGGCAACACTTAAGAGGGATAAG  GCAGGTCGACTTAGTGATGGTGATGGTGATGTGTTCTGTCTACAACTTCGCG  TAGAGTCGACATGAAATATGAATTTAATAATAGATTCCGAAC  GGAACTCTTTTACTCATAAAGAGCTCCTGATCATGTAGGTGTC  ATGATCAGGAGCTCTTTATGAGTAAAAGAGTTCCTAAAGATAGAGGTATTGATAATTCA  GCCTGCAGTTAGTGATGGTGATGGTGATGTTTTGTACGGTCGATATTCACCCTTATATTTTC  TAGAGTCGACATGCAAATAAACCGCCGAGG  TTAGGAACTCTTTTACTCATTGCTCCCTGGGCGTTG  AGGCCAACGCCCAGGGAGCAATGAGTAAAAGAGTTCCTAAAGATAGAGGTA  GCCTGCAGTTAGTGATGGTGATGGTGATGTTTTGTACGGTCGATATTCACCCT  CGAGCTCATGATTTTAGGCGTACCCAT  CTCTTTTACTCATCACCGTAGTGGGCACTGT  GCCCACTACGGTGATGAGTAAAAGAGTTCCTAAAGATAGAGGTATTGATAAT  TCCCCCGGGTTATTTTGTACGGTCGATATTC  ACGCGTCGACATGGCTCAGCGAAAACTGGCCTCTGTGA  ACTCTTTTACTCATAGCCACACCACCACTTGAG  TGGTGGTGTGGCTATGAGTAAAAGAGTTCCTAAAGATAGAGGTATTGATAAT  AACTGCAGTTATTTTGTACGGTCGATATTCAC  ACGCGTCGACATGAAGCTTTCACACCGCA  GGAACTCTTTTACTCATGGAAGAGAAGTTGGAGGACAGCT  CTCCAACTTCTCTTCCATGAGTAAAAGAGTTCCTAAAGATAGAGGTATTGATA  AACTGCAGTTATTTTGTACGGTCGATATTCAC  ACGCGTCGACATGGAAAACGTTTTGGAATTC  CTCTTTTACTCATGCCAACCAGACCGATGAGG  CGGTCTGGTTGGCATGAGTAAAAGAGTTCCTAAAGATAGAGGTATTGATAAT  AACTGCAGTTATTTTGTACGGTCGATATTCAC  ATCCTCTAGAGTCGAATGATTTTAGGCGTACCCATTCAATATTTGC  CCAAGCTTGCATGCCTTATTTTGTACGGTCGATATTCACCCTTATATTTTCAAT  ACGCGTCGACATGGCTCAGCGAAAACTGGCCTCTGTGA  AACTGCAGTTATTTTGTACGGTCGATATTCAC  ACGCGTCGACATGAAGCTTTCACACCGCA  AACTGCAGTTATTTTGTACGGTCGATATTCAC  ACGCGTCGACATGGAAAACGTTTTGGAATTC  AACTGCAGTTATTTTGTACGGTCGATATTCAC | XbaI  SalI  XbaI  SalI  SalI  PstI  SalI  PstI  SacI  SmaI  SalI  PstI  SalI  PstI  SalI  PstI  SalI  PstI  SalI  PstI  SalI  PstI |
| aar-F  aar-R  ado-F  ado-R  pntAB-F  pntAB-R  udhA-F  udhA-R  sgdh-F  sgdh-R  aasS-F  aasS-R  fdh1-F  fdh1-R  oleT_JE_-F  oleT_JE_-R  oleT_MC_-F  oleT_MC_-R | CCGGAATTCATGTTTGGCCTGATTGGCC  GCTCTAGATTAAATCGCCAGCGCCAGC  CGAGCTCATGCCGCAGCTGGAAGC  GCTCTAGATTACACCGCCGCCAGGCCATAC  CGAGCTCATGCGAATTGGCATACCAAG  GCTCTAGATTACAGAGCTTTCAGGATTGCA  CGAGCTCATGCCACATTCCTACGATTAC  GCTCTAGATTAAAACAGGCGGTTTAAACC  CCGGAATTCATGCCTGCCCCTTACAAAGA  GCTCTAGATTACGAGGACCAGTTGTTTTCG  ACGCGTCGACATGAACCAGTATGTGAACGA  AACTGCAGTTATTACAGATGCAGTTTACGC  CCGGAATTCATGTCGAAGGGAAAGGTTTTGCT  GCTCTAGATTATTTCTTCTGTCCATAAGCTCTGGTG  ACGCGTCGACAAGGAAGGCGCTGAAATG  AACTGCAGCATCATGCGTTGTACCATAAAC  ACGCGTCGACATGAGTAAAAGAGTTCCTAAAGATAG  AACTGCAGTTATTTTGTACGGTCGATATTC | EcoRI  XbaI  SacI  XbaI  EcoRI  XbaI  EcoRI  XbaI  EcoRI  XbaI  SalI  PstI  EcoRI  XbaI  SalI  PstI  SalI  PstI |
